# Reflections on our role as patient representatives for *CKJ*

**DOI:** 10.1093/ckj/sfaf202

**Published:** 2025-06-25

**Authors:** Takamasa Iwakura, Brooke M Huuskes

**Affiliations:** First department of Medicine, Division of Nephrology, Hamamatsu University School of Medicine, Hamamatsu, Japan; Department of Microbiology, Anatomy Physiology and Pharmacology, Centre for Cardiovascular Biology and Disease Research, La Trobe Institute for Molecular Science, School of Agriculture, Biomedicine and Environment, La Trobe University, Bundoora, VIC, Australia

We are deeply honored to serve as patient representatives for *CKJ* and would like to express our sincere gratitude to the Editorial Board for this opportunity. It is a privilege to contribute to a journal so dedicated to advancing kidney care through rigorous scientific inquiry and multidisciplinary collaboration.

My name is Takamasa Iwakura, an assistant professor in the Division of Nephrology at Hamamatsu University School of Medicine in Japan. I have lived with kidney disease since childhood and continue to receive treatment for chronic kidney disease. Simultaneously, I have been practicing as a nephrologist for over two decades, caring for patients across a wide spectrum of kidney conditions. This dual perspective—as both a long-term patient and a healthcare provider—offers me a unique vantage point from which to understand the clinical and personal dimensions of kidney disease. I applied for this role with the hope of further deepening my ability to view kidney disease through the lens of the patient. Although my clinical background provides valuable insights, I believe that maintaining a truly patient-centered perspective requires ongoing reflection, humility and learning.

My name is Brooke. I work as a Lecturer in Anatomy and run the Cardiorenal Disease Research Group at La Trobe University in Melbourne in Australia. My passion for research started after I had my kidney transplant in 2010. I always enjoyed science and wanted to give back to the community that gave me so much. Having my transplant allowed me to complete my honours degree and then a PhD in kidney regeneration. I have competed in the World Transplant Games and through this have been lucky to meet so many people from across the globe with shared lived experiences. But the thing I am most grateful for is that having my transplant allowed me to have a child. I feel I bring a unique perspective to this role, as a woman, as a mother and as a transplant recipient. I truly believe the statement ‘nothing about us, without us’ and aim to ensure patients are authentically included in research, especially when it directly affects their clinical experience or outcomes.

As patient representatives, we aim to contribute to *CKJ*’s mission in three main ways. First, by offering commentary on selected articles from the patient's perspective, we hope to highlight how research findings might be perceived by those directly affected by them. Second, we will contribute feedback during the peer-review process when appropriate, with an emphasis on aspects of care that may be underrepresented in clinical research but are of critical importance to patients. Third, we hope to offer broader insights into what patients value most in the context of kidney care and research, thereby helping to bridge the gap between clinical priorities and patient needs. Through these contributions, we aim to support *CKJ* in its ongoing efforts to incorporate patient voices into academic discourse. We look forward to learning from the Editorial Board and working collaboratively to ensure that the journal continues to reflect the experiences and perspectives of those living with kidney disease.

